# Reduced Graphene Oxide-Based Impedimetric Immunosensor for Detection of Enterotoxin A in Milk Samples

**DOI:** 10.3390/ma13071751

**Published:** 2020-04-10

**Authors:** Giovanna S. Rocha, Martin K. L. Silva, Ivana Cesarino

**Affiliations:** Department of Bioprocesses and Biotechnology, School of Agriculture, São Paulo State University (UNESP), Botucatu 18610-034, Brazil; giovanna.s.rocha@unesp.br (G.S.R.); martin.leme@unesp.br (M.K.L.S.)

**Keywords:** reduced graphene oxide, enterotoxin A, *Staphylococcus aureus*, impedimetric immunosensor

## Abstract

A simple, cheap, and less aggressive immobilization procedure for biomolecules using reduced graphene oxide (rGO) was employed to prepare an impedimetric immunosensor for detection of staphylococcal enterotoxin A (SEA) from *Staphylococcus aureus* in milk samples. The scanning electron microscopy, cyclic voltammetry, and electrochemical impedance spectroscopy (EIS) were used to monitor the single steps of the electrode assembly process. The glassy carbon (GC)/rGO platform detected the antigen-antibody binding procedures of SEA with concentrations of 0.5 to 3.5 mg L^−1^ via impedance changes in a low frequency range. The impedimetric immunosensor was successfully applied for the determination of SEA in milk samples.

## 1. Introduction

Foodborne Diseases are the resulting infections or intoxications from ingestion of contaminated food or water with chemicals or microorganisms, contributing with the growth of public health problems all around the world [1]. *Staphylococcus aureus* is a bacterium presented in approximately 25%–40% of the healthy human population and it is the etiologic agent of bovine mastitis, secreting toxins that causes immune reactions which characterizes food poisoning, whose symptoms are very similar to those of others infections or intoxications caused by other pathogens [2,3,4]. Among the enterotoxins it secretes, the staphylococcal enterotoxin A (SEA) is the one most associated with these intoxications, followed by the type B (SEB) and D (SED) because they are active even in small amounts and resistant to inactivation through gastrointestinal proteases, which allows it to pass through the intestinal epithelium, in addition to be thermoresistant [5].

The main techniques used to detect SEs include the enzyme immunoassay ELISA (enzyme-linked immunosorbent assay), Optimum Sensitivity Plate (OSP), and Polymerase Chain Reaction (PCR), adapted to meet the needs involving time spent for analysis and specificity and sensitivity for detection. Even so, these methods require at least 24 h to 72 h for each analysis and sample pre-treatments in order to reduce interference caused by agents contained in the samples themselves, and in the case of PCR, the detection of enterotoxin coding genes does not imply their presence in the sample [6,7].

In this context, the development of biosensors as alternatives for the analysis allows synthesizing the necessary requirements that the presented techniques cannot meet, such as fast response time, without the need for sample treatment, as well as high sensitivity and reproducibility [8]. Biosensor can be defined as being a sensor that uses biological materials, such as enzymes, antibodies, cells, tissues, among others that are connected to a device capable of transforming a biological signal into an electrical one. For the construction of a biosensor, it is necessary to immobilize the biological material on a surface, which depends on the characteristics of this material used for the interaction with the analyte of interest [9]. Recent studies have demonstrated the development of biosensors to detect staphylococcal enterotoxins, which contribute to reinforce the importance of such technology and help the development of new detection techniques. In the literature, Pimenta-Martins et al. [10] reported on an amperometric immunosensor to detect SEA in food based on a self-assembly monolayer and protein A on gold electrodes; Salmain et al. [11] developed a label-free piezoelectric immunosensor for direct detection of SEA and Rasooly et al. [12] approach was a sandwich biosensor with two antibodies to be labeled as a real time biosensor.

Graphene has become one of the most promising materials in the development of new electrochemical devices, being widely used in the construction of biosensors. Presenting characteristics such as a flat laminar structure with the thickness of an atom, extreme hardness, resistance and elasticity, it is chemically reactive with several substances [13]. Once formed by only a single layer of carbon in a 2D network [14], this structure gives it properties such as high electrical and thermal conductivity, characteristics of paramount importance for the preparation of an electrochemical biosensor. The carbon allotropy causes the individual sheets that make up graphene to tend to clump together, due to the strong π-π interactions and van der Waals interactions, which impairs the properties required for it [15]. For this reason, physical or chemical processes are necessary to control both its re-agglomeration and dispersion, and to improve its sensitivity. Reduced graphene oxide (rGO), also referred to as functionalized graphene sheets or chemically reduced graphene oxide, generally has abundant structural defects [16,17] and functional groups [18], which are presented as advantages for electrochemical applications.

The choice of substances to be immobilized on the surface of an electrode, in order to detect analytes in a sample, must meet requirements such as non-inhibition of electronic properties when they interact, as well as having appropriate chemical characteristics to react with the analyte. Antibodies are proteins that have three main functional groups, such as primary amines, sulfuric groups, and carbohydrates, which allow their modification as markers, crosslink, or immobilization on the surface of interest. In the case of immobilization of antibodies for the construction of biosensors, called immunosensors, the use of reagents that favor their permanence on the work surface is of paramount importance for the development of this detection method. The most common functional group for the immobilization of antibodies is the amine group, of which an N-terminal group is present in each chain of the polypeptide and next to the chain of lysine residues [19].

The aim of this work was to develop an immunosensor based on reduced graphene oxide without any immobilization agents, which impacts straightly on the construction, final cost, and applicability of the immunosensor, to detect SEA in pasteurized milk samples. The immobilization of the antibodies was successful, such as their stability on the electrode surface and the detection of SEA, demonstrating rGO employability on biosensors field and contributing to advances on this area.

## 2. Materials and Methods

### 2.1. Reagents and Solutions

Anti-Staphylococcal Enterotoxin A (polyclonal) antibody produced in rabbit, Staphylococcal enterotoxin A from *Staphylococcus aureus*, Bovine Serum Albumine (BSA) and Graphene Oxide (4 mg mL^−1^) were purchased from Sigma-Aldrich (São Paulo, SP, Brazil). Sodium Dodecyl Sulfate (SDS), Sodium borohydride (NaBH_4_), Ethanol, Potassium phosphate monobasic (KH_2_PO_4_), Sodium phosphate dibasic (Na_2_HPO_4_) were analytical grade.

Solutions used in this experiment were prepared using ultra-pure water (resistivity ≥ 18 MΩ cm) obtained from PURELAB Option-Q-ELGA–VEOLIA.

### 2.2. Synthesis of Reduced Graphene Oxide

**Figure 1:**
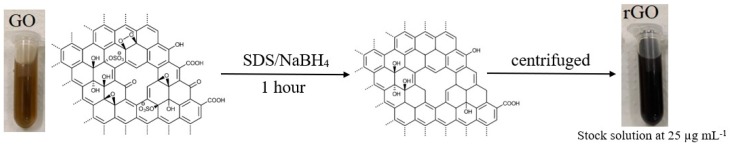
Schematic representation of reduced graphene oxide synthesis procedure.

The rGO was synthesized via a chemical reduction step approach. Precisely 20 mg of graphene oxide was dispersed in ethanol with 16.0 mg of SDS during 30 min in an ultrasonic bath (75% amplitude). The reduction step was conducted by adding 8.0 mg of NaBH_4_ into the mixture and a further 30 min sonication was performed. The obtained rGO was cleaned by centrifuging the synthesis and discharging the supernatant, this step was repeated three times. A solution of 25.0 µg mL^−1^ of the nanocomposite in ultra-pure water was prepared and kept under refrigeration. The schematic representation of reduced graphene oxide is shown in Figure 1.

### 2.3. Construction of the Impedimetric Immunosensor

**Figure 2:**
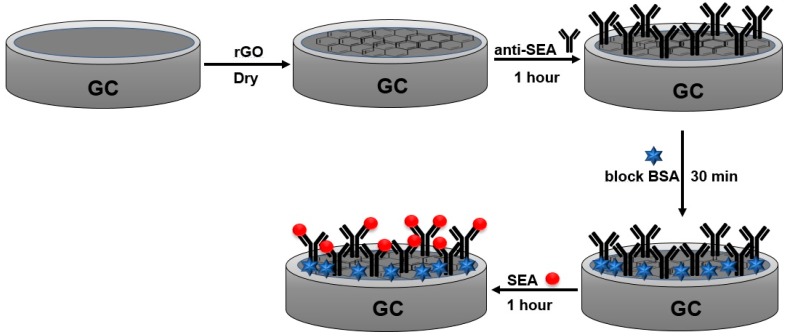
Schematic representation of the SEA immunosensor fabrication on a GC electrode.

The construction of the immunosensor was conducted using the following steps:

- The glassy carbon (GC) electrodes were polished using alumina slurries (Al_2_O_3_) and subsequently sonicated in ethanol and ultra-pure water. An aliquot of 10 µL of the rGO suspension was cast on the surface of the cleaned GC and dried at room temperature.
- Then, the electrode was incubated for 1 h with a 10 µL aliquot of the Anti-Staphylococcal Enterotoxin A antibody (anti-SEA) solution (0.10 mg mL^−1^). The weakly bonded antibody molecules were washed with a PBS (0.2 mol L^−1^, pH 7.4) solution for 30 s under soft stirring.
- The residual sites on rGO surface that may lead to non-specific binding of antigen molecules were blocked by incubating the sensor with 10 µL of a bovine serum albumin solution (1%, BSA), during 30 min, and washed again with the PBS solution for removal of non-bonded BSA. Then, the sensor was ready for measuring of Staphylococcal enterotoxin A (SEA) antigen. The schematic representation of the immunosensor fabrication is shown in Figure 2.

### 2.4. Scanning Electron Microscopy

Surface morphology for all the nanomaterials was characterized using a scanning electron microscopy (FEG−SEM) and the images were recorded using a model Quanta 200 (FEI Company, Hillsboro, OR, USA) localized in the Electron Microscope Center of the Institute of Biosciences of Botucatu, UNESP (CME-IBB-UNESP).

### 2.5. Cyclic Voltammetry (CV) and Electrochemical Impedance Spectroscopy (EIS) Experiments

All electrochemical measurements were conducted in a PGSTAT-128N Autolab electrochemical system (Metrohm, Utrecht, The Netherlands). A three-electrode cell was set as follows: a glassy carbon (GC) as a working electrode, a platinum plate as an auxiliary electrode and Ag/AgCl (3.0 mol L^−1^) as the reference electrode. The electrochemical measurements were conducted in a 0.2 mol L^−1^ PBS (pH 7.4) solution containing 0.1 mol L^−1^ of KCl and 5.0 mmol L^−1^ of the redox couple Fe(CN)_6_]^3−/4−^. Cyclic voltammograms scans were recorded in the potential range of −0.3 to 0.9 V *vs.* Ag/AgCl, with a scan rate of 50 mV s^−1^.

The EIS experiments were recorded in open circuit potential (OCP), with frequency ranging from 10^7^ to 10^−2^ Hz, amplitude of 10 mV with 10 points per decade using the FRA32M module coupled with the potentiostat. All EIS results were fitted in equivalent circuit using the Electrochemical Circle Fit on NOVA 2.1 software (version 2.1, Metrohm, Utrecht, The Netherlands), and the obtained charge transfer resistance (*R*_ct_) draw from the impedance data was used for obtaining a quantitative signal of SEA antigen concentration in the assay.

### 2.6. Analysis of Milk Samples

The immunosensor response was tested in a 1: 100 dilution sample of whole pasteurized milk (in 0.2 mol L^−1^ PBS buffer, pH 7.4) by incubating the electrode in SEA solutions at different concentrations (prepared by dilution of SEA stock solution in 0.2 mol L^−1^ PBS solution, pH 7.4). Subsequently, the immunosensor was washed carefully with ultra-pure water, the EIS measurements were performed, and the *R*_ct_ was analyzed.

## 3. Results and Discussion

### 3.1. Morphological Characterization of GO and rGO Nanocomposites

**Figure 3:**
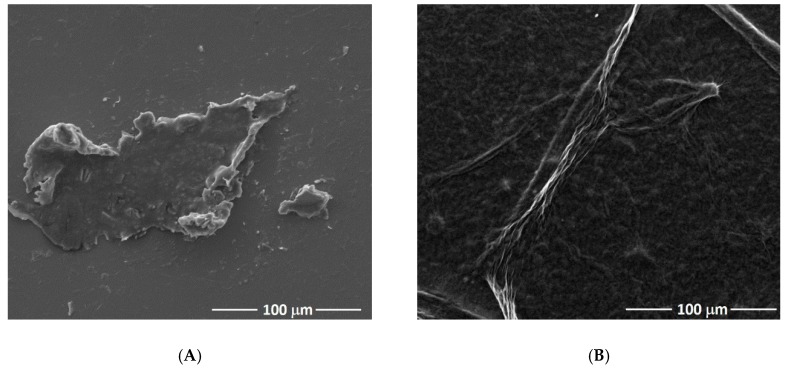
SEM micrographs for GO (**A**) and rGO (**B**) materials.

Scanning electron microscopy was used to characterize the morphology of the nanomaterials. Figure 3A shows a typical graphene oxide sheet that tends to form single sheets of graphene through π-π interaction and stacking of GO sheets. After the chemical reduction step, the sheet surface is twisted and wrinkled, and with undulations as shown in Figure 3B.

### 3.2. Electrochemical Characterization of the Immunosensor

**Figure 4:**
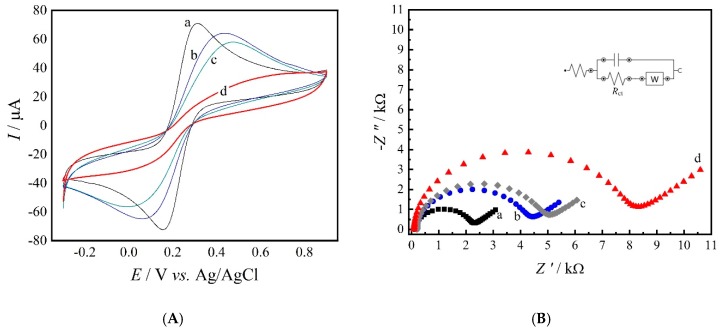
Cyclic voltammograms (**A**) and Nyquist plots (**B**) for: (a) GC/rGO, (b) GC/rGO/anti-SEA, (c) GC/rGO/anti-SEA/BSA and (d) GC/rGO/anti-SEA/BSA/SEA. Both experiments were performed in a 0.2 mol L^−1^ PBS (pH 7.4) solution containing 0.1 mol L^−1^ of KCl and 5.0 mmol L^−1^ of Fe(CN)_6_]^3−/4−^. *Inset*: equivalent circuit.

Figure 4A,B shows the CV and EIS, respectively, electrochemical characterizations of each step of the immunosensor fabrication. The cyclic voltammogram obtained for the GC/rGO electrode (curve a) showed a well-defined profile in the presence of the redox couple, with oxidation and reduction processes at +312 mV and +156 mV, respectively, as shown in Figure 4A. As the construction of the immunosensor was carried on, it is possible to identify a decrease in the anodic and cathodic peak currents of the redox couple and an increase in Δ*E*_p_ (the Δ*E*_p_ values were 156 mV for the GC/rGO electrode and 292 mV after antibody immobilization). These results indicate that in each step a biomolecule (antibody, BSA, or antigen) is bonded to the electrode surface, which interferes in the electron transfer processes of the redox couple. As expected, the profile for the GC/rGO/anti-SEA/BSA/SEA suffered a great change in the reversibility of the system, showing almost no redox processes.

Figure 4B displays the Nyquist plots for the assay indicating an increase in the charge transfer resistance (*R*_ct_) values during the immunosensor fabrication. As expected, an *R*_ct_ of 2.14 kΩ was found for the GC/rGO electrode (curve a). This result is associated with rGO’s remarkable electron transfer properties, which facilitates electrons transfer kinetics [20]. However, biomolecules, such as antibodies, antigens, and enzymes, have poor electrical conductivity at low frequencies and may difficult the electron transfer process [21,22].

Antibody molecules can be immoblized through several procedures such as N-ethyl-N-(3-dimethylaminopropyl) carbodiimide/N-hydroxysuccinimide (EDC/NHS) [23], gold nanoparticles [24], amino-terminated perylene derivative (PTCNH_2_) [25], self-assembled monolayers [26], thiolated surfaces [27], which all are meant to provide site-directed immobilization and prevent protein denaturation [28].

The reduction step proposed in this experiment is intended to decrease the oxygen content on the nanomaterial’s surface, as well, the insulating properties of GO [29]. However, it has been reported that NaBH_4_, as the reducing agent, is efficient in the reduction of carbonyl groups, but has a moderate efficacy in reducing epoxy, carboxylic acids, and alcohol groups. These other functional groups may be present on rGO surface after the reduction step [30,31]. This characteristic of the rGO nanocomposite may explain the successful immobilization of anti-SEA molecule with the remaining carboxylic groups, without using any bioconjugation agent. In addition, previous works reported the efficienty immobilization of enzymes in rGO modified electrodes [32,33,34,35].

Therefore, GC/rGO/anti-SEA (curve b) electrode showed an *R*_ct_ value of 4.15 kΩ, which shows the antibody immobilization on the electrode surface.As expected, after BSA incubation (curve c), an *R*_ct_ of 4.86 kΩ was found due to BSA bonding to unspecific sites on the immunosensor. Finally, after the antigen incubation step, the GC/rGO/anti-SEA/BSA/SEA showed a *R*_ct_ of 8.12 kΩ in the presence of 10 µL of the antigen (3.0 mg L^−1^).

Hence, both CV and EIS characterizations indicated that the immunosensor can successfully be used to monitor the antigen-antibody binding procedures of SEA.

### 3.3. Optimization of Anti-SEA Immobilization and Stability

**Figure 5:**
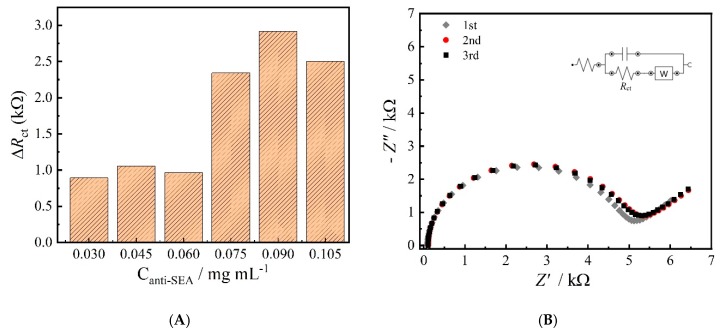
Responses of the proposed immunosensor to a fixed amount of SEA using different concentrations of antibody ranging from 0.030 to 0.105 mg mL^−1^ (**A**) and successive measurements of the sensor with immobilized antibody (**B**).

EIS experiments were performed to evaluate the amount of anti-SEA antibody necessary for the construction of the immunosensor. Therefore, five different concentrations of antibody were exposed to a fixed amount of 3.0 mg L^−1^ of the antigen (SEA) to obtain maximum response for the toxin level. Figure 5A shows de ∆*R*_ct_ values obtained for each assay, which demonstrates an increase in the resistance value, reaching its maximum with antibody concentration at 0.090 mg mL^−1^ for the immunosensor construction. The ∆*R*_ct_ values were obtained from a simple subtraction of the resistances obtained before and after antigen incubation in each assay. Hence, the concentration of 0.090 mg mL^−1^ was used for the subsequent experiments.

To evaluate the stability of the immobilized antibody on the GC/rGO platform, three consecutive EIS experiments were conducted on the sensor and between each measurement the electrode was rinsed with a 0.2 mol L^−1^ PBS (pH 7.4) buffer solution. The *R*_ct_ values did not showed significant different between the measurements, with a standard deviation of 4.61% (*n* = 3). In addition, the Nyquist plots of this experiment were very similar, indicating that the antibody molecules were strongly bonded to the GC/rGO platform as shown in Figure 5B.

### 3.4. Detection of Staphylococcal Enterotoxin A (SEA) Using the Impedimetric Immunosensor

**Figure 6:**
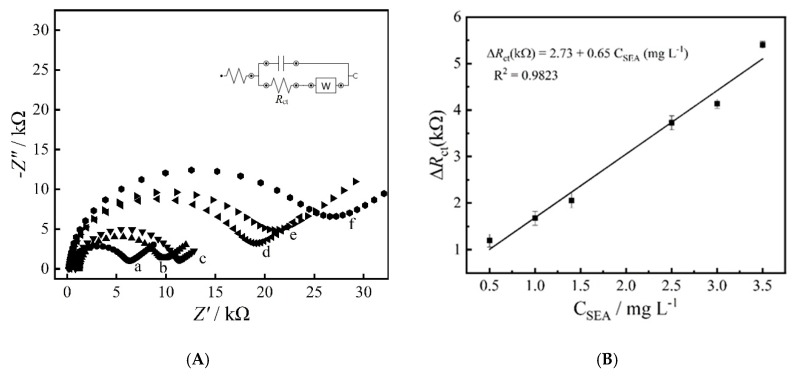
Nyquist plots of the immunosensor incubated with different SEA concentrations (**A**) and respective calibration curve from 0.5 to 3.5 mg L^−1^ (**B**).

The analytical performance of the impedimetric immunosensor was evaluated by using the Nyquist plots obtained from the EIS experiments at different concentrations of SEA as shown in Figure 6A, which demonstrates that the *R*_ct_ value increases as the antigen concentration increased. The calibration curve for the detection of SEA antigen under optimized conditions is shown in Figure 6B. A linear relationship (R^2^ = 0.9823) between the Δ*R*_ct_ (subtraction of electrode’s *R*_ct_ before and after antigen incubation) and concentration of SEA was obtained in the range of 0.5 and 3.5 mg mL^−1^. The obtained limit of detection of 0.102 µg mL^−1^ (calculated as Limit of Detection (LOD) = 3 SD / Slope) and a limit of quantification of 0.330 µg mL^−1^ (calculated as LOQ = 10 SD / Slope).

Table 1 summarizes a comparison of different methodologies and respective limits of detection toward SEA determination. Upadhyay et al. [36], investigated the SEA concentration levels in milk samples using a lateral flow immunoassay (LFIA) coupled with gold nanoparticles (AuNPs) with a limit of detection (LOD) of 0.5 µg mL^−1^. In addition, Haddada et al. [37] also investigated the properties of AuNPs for SEA detection using a localized surface plasmon resonance (LSPR), presenting a LOD of 0.005 µg mL^−1^. Regarding electrochemical immunosensors, Salmain et al. [11] developed a label-free immunosensor using a self-assembled monolayer of cysteamine onto gold electrodes and a quartz crystal microbalance with dissipation (QCM-D) as transduction method. The sensor showed a good linear response in the range of 50–1000 ng mL^−1^ with a LOD of 20 ng mL^−1^.

Pimenta-Martins et al. [10] developed an amperometric immunosensor for SEA detection in cheese samples using an Au electrode modified with protein A for anti-SEA antibody immobilization. The chronoamperometric measurements showed a good linear response in the range of 0.016 to 0.150 mg mL^–1^ of SEA, with a LOD of 33.9 ng mL^−1^. Additionally, AuNPs and Au electrodes were extensively used for electrochemical immunosensors construction due to simple fabrication procedures, easy functionalization, and formation of self-assembled monolayers [38].

### 3.5. Real Sample Analysis

The performance of the immunosensor in milk samples was evaluated by the standard addition method. Pasteurized milk purchased from local stores was diluted (1: 100) in a 0.2 mol L^−1^ PBS pH 7.4 solution and the sample was spiked with SEA giving a final concentration of 2.0 mg L^−1^. After first measurement, the sample was spiked with 0.5, 1.0 and 1.5 mg L^−1^ of the SEA standard solution. The electrodes with immobilized anti-SEA antibodies were incubated in these aliquots for 30 min at room temperature (20 °C), and EIS measurements were performed. The results are summarized in Table 2. The immunosensor showed a good response toward SEA determination in the milk sample, with recoveries between 95 and 108%.

## 4. Conclusions

This work presented the fabrication of a label-free impedimetric immunosensor based on an GC/rGO platform without the need for any bioconjugation agents, polymers, cross-linking materials, and complicated procedures. The developed immunosensor was able to detect selectively SEA levels with a limit of detection of 0.102 µg mL^−1^. The real sample analysis demonstrated the robustness of the sensor toward SEA determination in milk samples, showing good specificity and reproducibility.

## Figures and Tables

**Table 1:** Limit of detection comparison of different biosensors for SEA detection.

Methodology	Materials	LOD (µg mL^−1^)	Ref.
LFIA ^1^	Gold nanoparticles	0.500	[36]
LSPR ^2^	Gold nanoparticles	0.005	[37]
Piezoelectric immunosensor	Cysteamine on Au electrodes	0.020	[11]
Amperometric immunosensor	SAM ^3^ on Au electrode	0.339	[10]
Impedimetric immunosensor	Reduced Graphene Oxide/GC	0.102	This work

^1^ Lateral flow immunoassay (LFIA). ^2^ Localized surface plasmon resonance (LSPR). ^3^ Self-assembly monolayer.

**Table 2:** Results for the determination of SEA in pasteurized milk samples by impedimetric proposed method.

Repetition	Added (mg L^−1^)	Determined (mg L^−1^)	Relative Errors (%)
1	2.0	1.90	−4.80
2	2.0	2.05	2.70
3	2.0	2.16	8.25
Mean ± SD		2.04 ± 0.13	
